# Effect of Heat Treatment Process on the Mechanical Properties of 40MnBNbVTiMo Hot-Stamped Steel and Industrial Validation

**DOI:** 10.3390/ma19183941

**Published:** 2026-09-17

**Authors:** Dongrui Sun, Ying Li, Guangxiang Cao, Ziming Tang

**Affiliations:** 1 Institute of Advanced Structure Technology, Beijing Institute of Technology, Beijing 100081, China; sundongrui@faw.com.cn; 2 State Key Laboratory of Advanced Vehicle Integration and Control, China FAW Group Co., Ltd., Changchun 130013, China; caoguangxiang@faw.com.cn (G.C.); tangziming3@faw.com.cn (Z.T.)

**Keywords:** hot-stamped steel, microalloying, precipitate, ultra-high strength

## Abstract

**Highlights:**

- CCT curves of 40MnBNbVTiMo steel are established.
- The optimal heat treatment process is heating to 910 °C for 5 min.
- After hot stamping, the 40MnBNbVTiMo steel forms (Nb,Ti) C and (Nb,Ti,V) C precipitates, which play a role in grain refinement and precipitation strengthening.

**Abstract:**

This study developed a novel hot-stamped steel 40MnBNbVTiMo for automotive application, which achieves ultra-high strength by microalloying and hot stamping process optimization. The continuous cooling transformation (CCT) curve is established, revealing that *A*_c1_, *A*_c3_, *M*_s_, and *M*_f_ are 765.2 °C, 812.2 °C, 314 °C, and 190 °C, respectively, and the martensitic critical cooling rate is approximately 1 °C/s. The effects of the austenitizing temperatures (890 °C, 910 °C, 930 °C) on the microstructure and mechanical properties are systematically examined. Optimal performance is achieved at 910 °C for 5 min, with a tensile strength of 2201 MPa, an elongation of 6%, and refined prior austenite grains (7.47 µm, grain size level 11.4). TEM results showed a martensitic matrix with high dislocation density and uniformly dispersed (Nb,Ti) C and (Nb,Ti,V) C precipitates, ranging from 75 nm to 400 nm, which provide strong precipitation strengthening and grain refinement. The mechanical performance and the optimized heat treatment process are validated through numerical simulations and forming tests conducted on the door anti-collision beam part; the mechanical properties of *R*_p0.2_, *R*_m_, and *A*_t_ are 1480 MPa, 2287.9 MPa, and 4.96%, respectively.

## 1. Introduction

Against the backdrop of the “dual carbon goals”, the automotive industry has an even greater need for lightweighting [1,2]. Meanwhile, with the continuous improvement in automobile safety standards, there is an urgent demand for higher strength levels in hot-stamped steel [3]. The development of hot-stamped steel materials conforms to the industry’s development trend, with the strength grade advancing from the commonly used 1500 MPa to the 1800 MPa~2000 MPa strength grade [4,5,6]. Generally, enhancing the strength property in hot-stamped steel primarily involves three methods. First, increasing the carbon content is a primary means of enhancing strength. For instance, the carbon content of 1500 MPa grade hot-stamped steel is about 0.22%~0.24%, while the carbon content of 1800 MPa grade hot-stamped steel is about 0.30%~0.31%, and the carbon content of 2000 MPa grade hot-stamped steel, such as 38MnB5, is about 0.36%~0.38% [7,8,9]. This is because the number of dislocations in the hot-stamped steel increases with the rising carbon content, resulting in a more pronounced dislocation strength effect [10]. However, based on the classical inverse relationship between strength and ductility in high-strength steel, increasing strength inevitably reduces ductility and toughness. Higher carbon content also degrades weldability, thereby limiting the application of hot-stamped steel [11]. Secondly, various studies have demonstrated that microalloying technology provides an effective solution to the inherent trade-off between strength and ductility in hot-stamped steel [12,13,14]. Additionally, microalloying technology also plays a positive role in enhancing the hydrogen embrittlement resistance of hot-stamped steel [15,16,17,18,19]. Microalloying elements such as Ti, Nb, V, and Mo are strong carbide-forming elements that can also segregate as solute atoms at austenite grain boundaries, thereby affecting the microstructure and properties of hot-stamped steels through precipitation-strengthening and solute drag effects [20,21]. Finally, optimizing production processes can enhance the mechanical properties of hot-stamped steel to some extent, especially since heat treatment plays a decisive role in tailoring these microstructural constituents [22,23,24,25,26]. As the strength of hot-formed steel increases, its ductility and toughness generally decrease, which creates a trade-off between strength and these properties. Additionally, there is a risk of hydrogen embrittlement. Consequently, when developing ultra-high-strength steel, a significant challenge is to achieve a balanced improvement in material properties while minimizing the risk of hydrogen embrittlement. It is also essential to ensure that the steel meets the requirements for applications such as welding and painting.

This paper designed a hot-formed steel 40MnBNbVTiMo through microalloying technology and systematically investigated the relationship among heat treatment processes, microstructure, and mechanical properties. The material developed in this work is primarily intended for safety-critical automotive structural components, such as door anti-collision beam, B-pillar, and tunnel reinforcement, which require ultra-high strength to resist impact loads while maintaining sufficient ductility to absorb crash energy. It is essential to provide references for the development and application of higher-strength grades of hot-stamped steels in automotive components.

## 2. Experimental Details

### 2.1. Materials and Thermal Expansion Experiments

The chemical composition of the hot-stamped steel 40MnBNbVTiMo is presented in Table 1. This steel incorporates multiple microalloying elements, including Nb, V, Ti, and Mo, which are strong carbide/nitride formers, which contribute to grain refinement and precipitation strengthening, and further enhance the hydrogen trapping capability. The dilatometry test is used to determine the critical cooling rate for hot stamping, according to the YB/T 5128-2018 standard [27]. The samples are machined into Φ4 mm × 10 mm and subjected to simulated static CCT testing on a DIL805A thermal expansion analyzer (Waters Corporation, Milford, MA, USA)The samples are heated to 1000 °C at a heating rate of 20 °C/s, held at that temperature for 300 s, and then cooled to room temperature at different cooling rates (0.05 °C/s, 0.1 °C/s, 0.5 °C/s, 1 °C/s, 5 °C/s, 10 °C/s, 20 °C/s, and 40 °C/s).

### 2.2. Heat Treatment and Mechanical Tests

**Figure 1:**
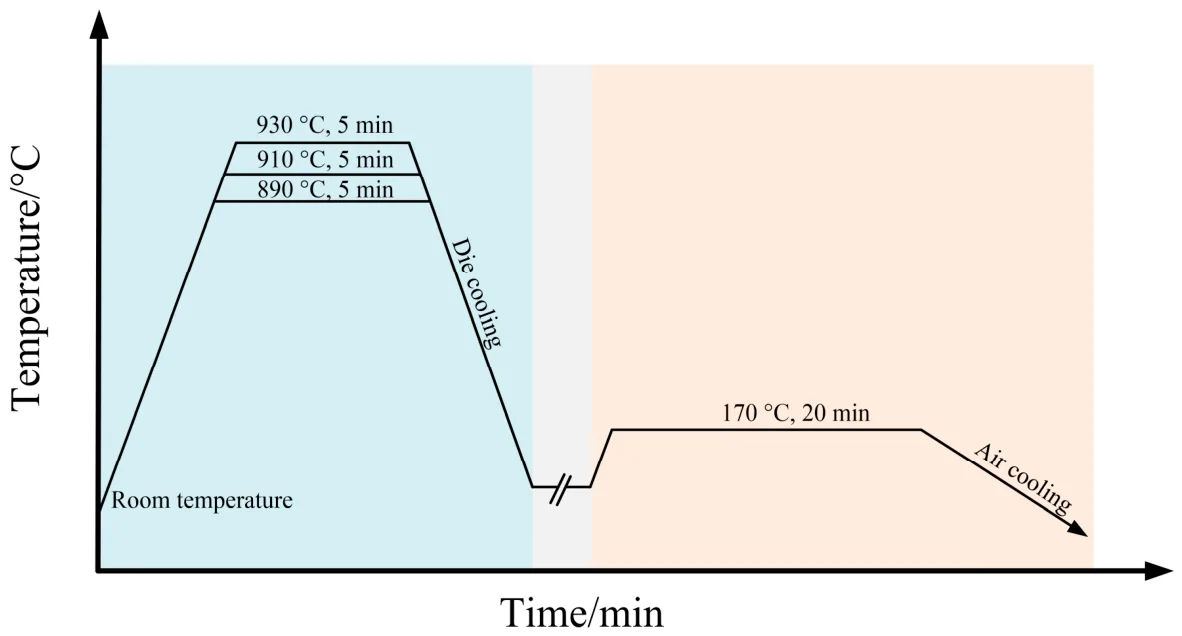
The schematic diagram of the heat treatment process.

Sheets with a final thickness of 1.8 mm are obtained through a route involving smelting, continuous casting, hot rolling, cold rolling, and batch annealing. The flat-die quenching is employed to investigate the effects of different austenitizing temperatures on the mechanical properties and microstructure of the 40MnBNbVTiMo steel. The sheet is machined into 300 mm × 230 mm × 1.8 mm, and heated to 890 °C, 910 °C, and 930 °C, with a 5 min hold at each temperature. After heating, the sheet is quickly transferred to a flat die with cooling water channels for stamping and quenching for 12 s. To evaluate the performance of the 40MnBNbVTiMo steel in an automotive body component, a baking test is conducted on the quenched sheet to simulate the baking process during vehicle body painting. The baking test is performed using an STPH-102 high-temperature oven (ESPEC Corporation, Osaka, Japan), and set to a heating temperature of 170 °C for 20 min. The heat treatment process for the sheet is illustrated in Figure 1.

**Figure 2:**
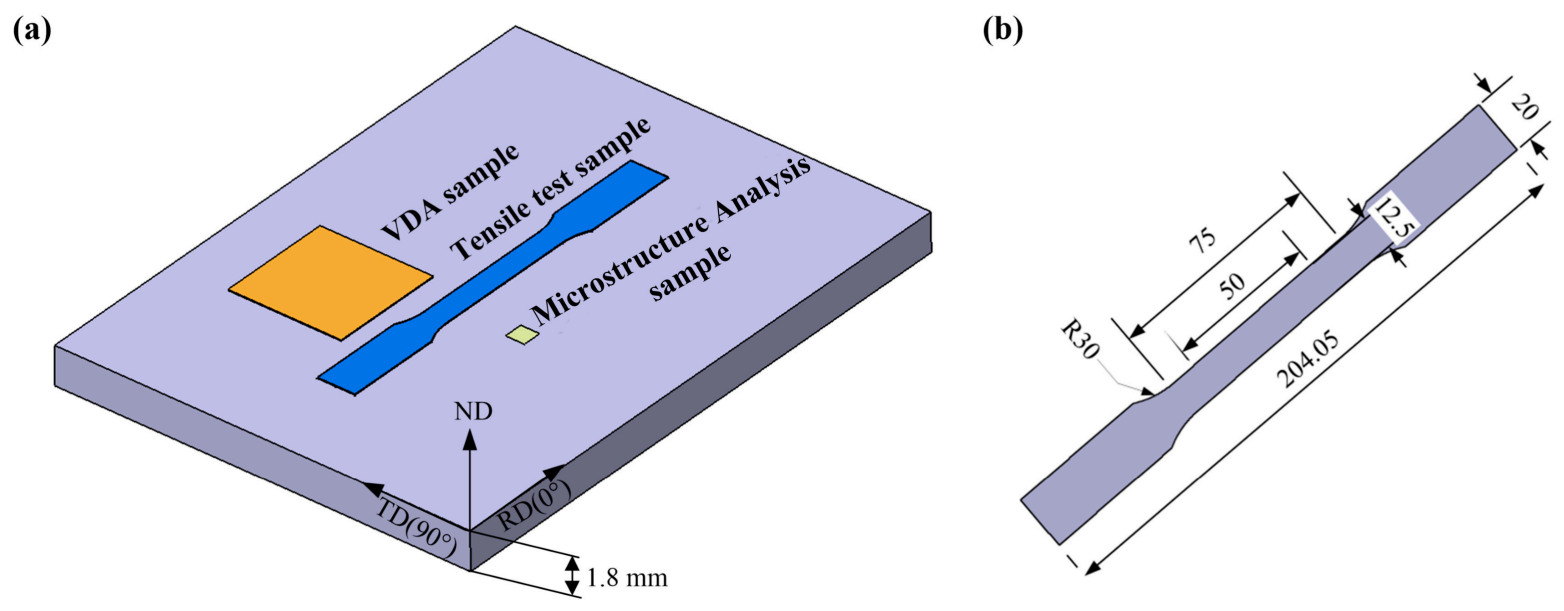
Sampling schematic diagram: (**a**) sample location, (**b**) tensile sample dimension (units: mm).

Samples for mechanical tests and microstructure analysis are machined from the heat treatment sheet, as shown in Figure 2a. Mechanical properties are identified by the uniaxial tensile test with the standard A50 samples (Figure 2b), according to the GB/T 228.1–2021 standard [28], with a gage width of 12.5 mm and a gage length of 50 mm, for which the tensile samples are vertical to the rolling direction. The uniaxial tensile test is performed by a JS160 electro-mechanical universal testing machine (Sinotest Equipment Co., Ltd., Changchun, China) with a tensile strain rate of 1 mm/min at room temperature. Given that hot-stamped steel parts are vital to the vehicle crash safety, evaluation of their bending properties is imperative to verify their in-service performance. The three-point bending tests are conducted on a Shimadzu AGX-V2 universal testing machine (Shimadzu Corporation, Kyoto, Japan) in accordance with VDA 238-100, using a 60 mm × 60 mm sample. The test employed a punch tip radius of 0.4 mm, a support roller spacing of 4.1 mm, and a punch speed of 20 mm/min. For each temperature condition, three samples are selected for the tensile test and three-point bending test, and the average mechanical properties are obtained.

### 2.3. Microstructural Characterization

Microstructural characterizations are performed using an optical microscope (OM, OLYMPUS BX53-P, Olympus Corporation, Tokyo, Japan), a scanning electron microscope (SEM, JSM-7900F, JEOL Corporation, Tokyo, Japan), electron backscatter diffraction (EBSD, JSM-7900F, JEOL Corporation, Tokyo, Japan), and transmission electron microscopy (TEM, JEOL 2100, JEOL Corporation, Tokyo, Japan). The observation surface is on the rolling direction–normal direction (RD-ND) plane. Samples for OM are polished and etched with a supersaturated aqueous solution of picric acid containing a small amount of sodium tris (phenyl) sulfonate in an 80 °C water bath for 15 min, during which the samples are periodically removed and wiped to reveal clearer grain boundaries. The grain size is measured using the intercept method according to the GB/T 6394–2017 standard [29]. Samples for SEM are mechanically polished and etched with a 4% nital acid-alcohol solution with 15 s; EBSD measurements (step size: 0.05 μm) are carried out after electrolytic polishing in an electrolyte solution (9% alcohol perchloric) at liquid nitrogen temperature. The acquired orientation data are processed using Channel 5 software. Grain boundaries with the misorientation of 2°~15° are classified as low-angle grain boundaries (LAGBs), and those above 15° are high-angle grain boundaries (HAGBs). The TEM samples are mechanically ground and thinned from 400 μm to 70 μm and then punched into small circular disks with a diameter of 3 mm. It is double-etched in a 5% perchloric acid alcohol, with the working temperature at −40 °C and the voltage at 50 V. In this study, the carbon extraction replication technique is utilized to extract the precipitated phases from the test steel and to characterize them. After mechanical grinding, polishing, and etching of the samples, carbon is sprayed on the surface. The carbon film is then peeled off using a 10% nitric acid alcohol, and the carbon film is retrieved using a copper mesh. At the same time, the composition of the precipitates is analyzed by EDS (Energy Dispersive Spectroscopy, Oxford Instrments, High Wycombe, UK). Phase analysis is performed using a Rigaku Ultima IV X-ray diffractometer (Rigaku Corporation, Tokio, Japan) with Cu-Kα radiation, operating at 35 kV and 25 mA. The scanning range (2θ) is from 30° to 120°, and the scanning speed is 2°/min.

## 3. Results and Discussion

### 3.1. CCT Curves of Test Steel

**Figure 3:**
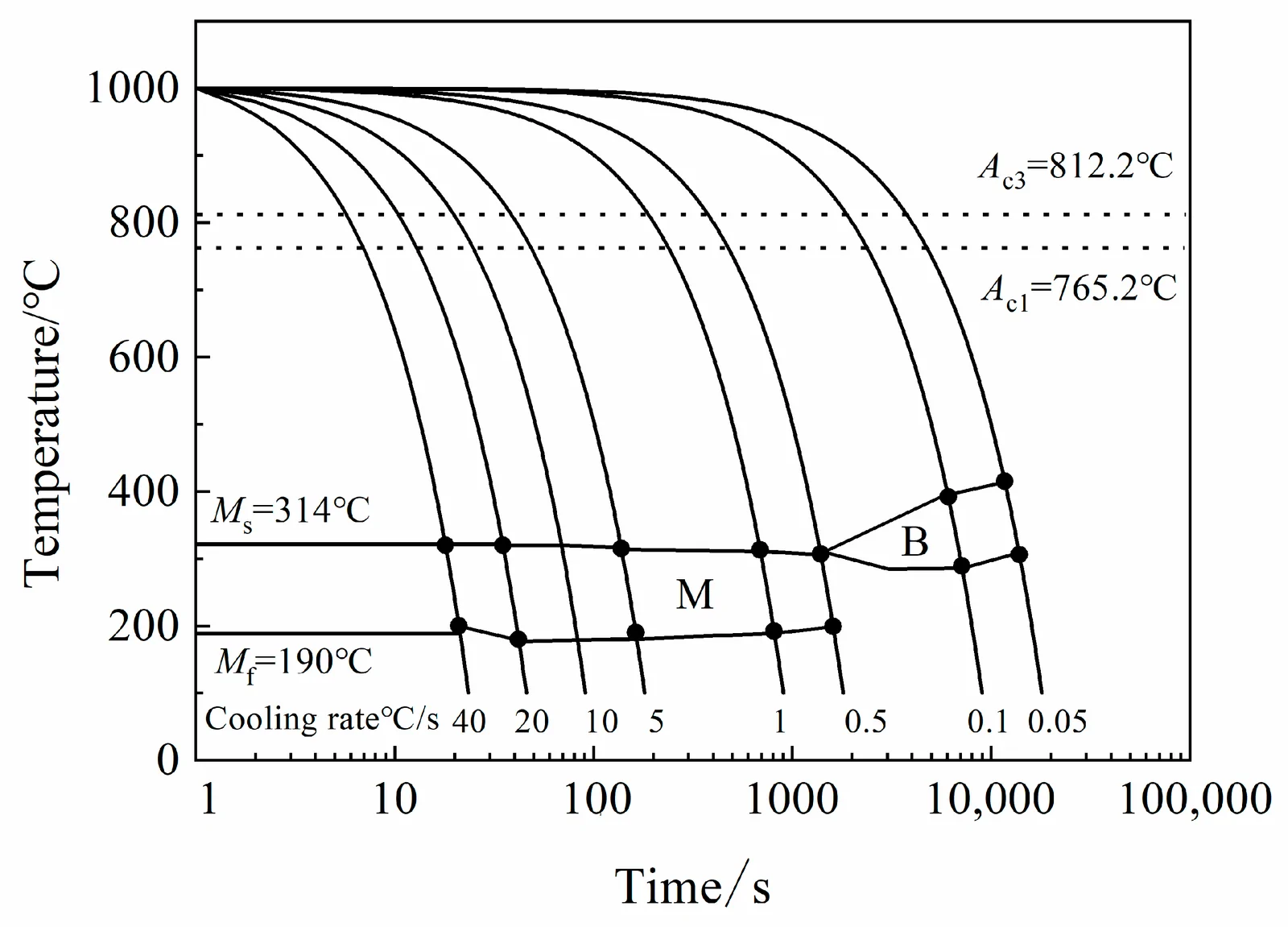
CCT curves of the test steel.

The CCT curves of the 40MnBNbVTiMo steel determined by microstructure and microhardness measurements are shown in Figure 3. During cooling, the test steel undergoes two main types of phase transformations, which are the bainite and martensite transformations. When the cooling rate is between 0.05 °C/s and 0.5 °C/s, the test steel primarily undergoes a bainitic transformation; when the cooling rate is 1 °C/s, the martensite start temperature (*M*_s_) and finish temperature (*M*_f_) are 314 °C and 190 °C, respectively, which indicate that the martensitic critical cooling rate is approximately 1 °C/s. The CCT curves provide a theoretical basis for developing a hot stamping process for 40MnBNbVTiMo steel.

### 3.2. Mechanical Results of the Test Steel

The mechanical properties after baking are shown in Table 2. The test results indicate that when the heating temperature is 890 °C, the material exhibits lower tensile strength. When the heating temperature is 910 °C or 930 °C, the strength values are relatively similar; however, the average elongation is higher at 910 °C. Also, the results show that the heating temperature significantly affects the cold bending performance. At 890 °C, the bending angle is about 37.9°, and it increases notably when the temperature reaches 910 °C or above. Specifically, the average ultimate bending angle at 910 °C is approximately 12.5% higher than that at 890 °C. Considering both tensile test results and bending test results, a heating temperature of 910 °C is recommended for the hot stamping process to achieve a favorable strength–toughness synergy.

### 3.3. Microstructure Analysis of the Test Steel

**Figure 4:**
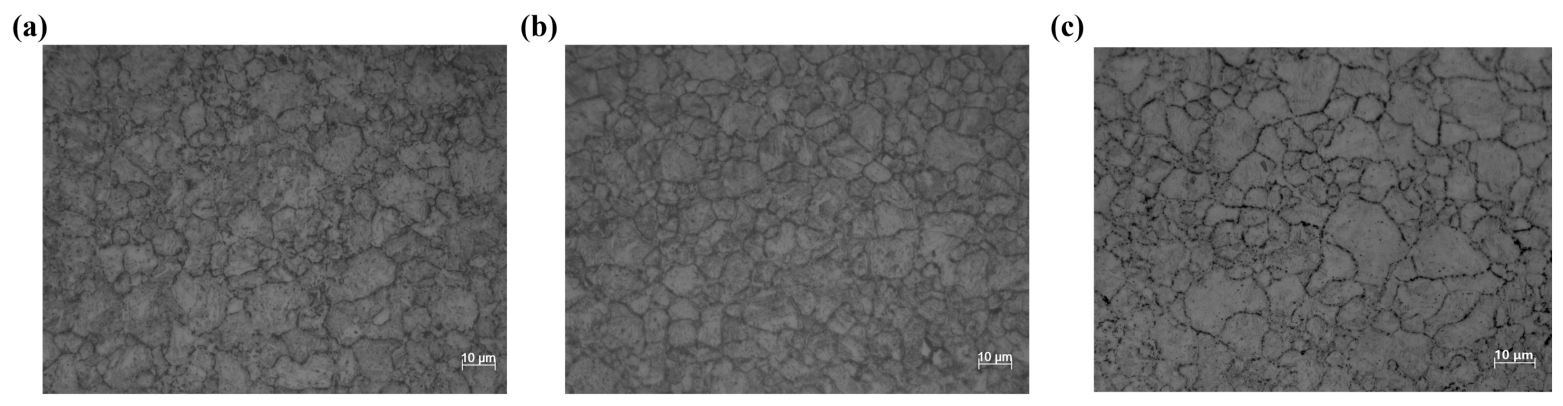
OM images of the prior austenite grain at different heating temperatures: (**a**) 890 °C, (**b**) 910 °C, (**c**) 930 °C.

The OM images of the prior austenite grain at different heating temperatures are shown in Figure 4. According to the intercept method, the grain size levels are 10.49, 11.14, and 10.72, respectively, with the average grain size of 9.38 μm, 7.47 μm, and 8.65 μm. The refinement of prior austenite grains in the test steel is primarily attributed to the Zener pinning effect, where the precipitation of microalloyed carbonitrides effectively impedes grain boundary migration [30]. As the austenitizing temperature increases from 890 °C to 910 °C, the average grain size decreases from 9.38 µm to 7.47 µm, accompanied by a more uniform grain distribution at the higher temperature. This refinement is primarily attributed to the presence of coarse and heterogeneously distributed (Nb,Ti,V) (C,N) precipitates at 890 °C, which exert non-uniform Zener pinning forces on grain boundaries, thereby allowing the preferential growth of certain grains. When the temperature is 910 °C, partial dissolution of metastable precipitates occurs, and the released microalloying elements reprecipitate in finer and more dispersed forms during the subsequent holding period, thereby enhancing the Zener pinning effect. Concurrently, solute segregation at grain boundaries contributes to a drag effect. These mechanisms synergistically suppress grain boundary migration. When the temperature exceeds 910 °C, thermal activation becomes dominant, and the driving force for grain boundary migration substantially outweighs the pinning resistance, leading to noticeable grain coarsening. Furthermore, the refined grains are beneficial for the synergistic improvement in strength, ductility, and toughness in the hot-stamped steel. Considering both grain size and uniformity, the optimal heating temperature under the present experimental conditions is determined to be 910 °C.

**Figure 5:**
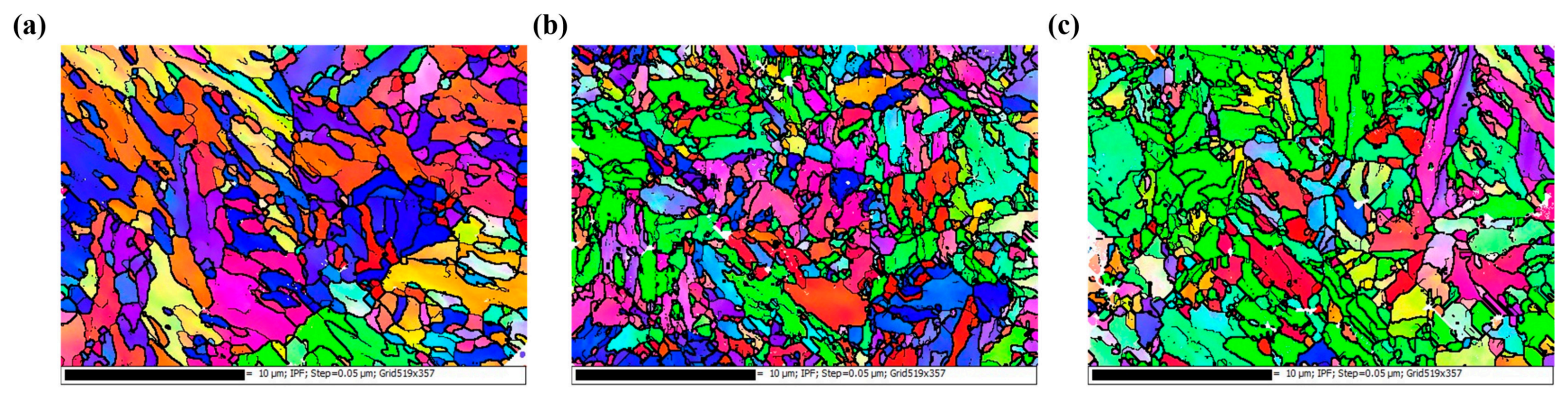
IPF maps of the test steel quenched at different heating temperatures: (**a**) 890 °C, (**b**) 910 °C, (**c**) 930 °C.

Figure 5 presents the IPF maps of the test steel quenched at different austenitizing temperatures, where distinct colors correspond to martensitic blocks with differing crystallographic orientations. The average martensitic blocks sizes are measured as 1.36 μm, 0.78 μm, and 1.01 μm, respectively.

**Figure 6:**
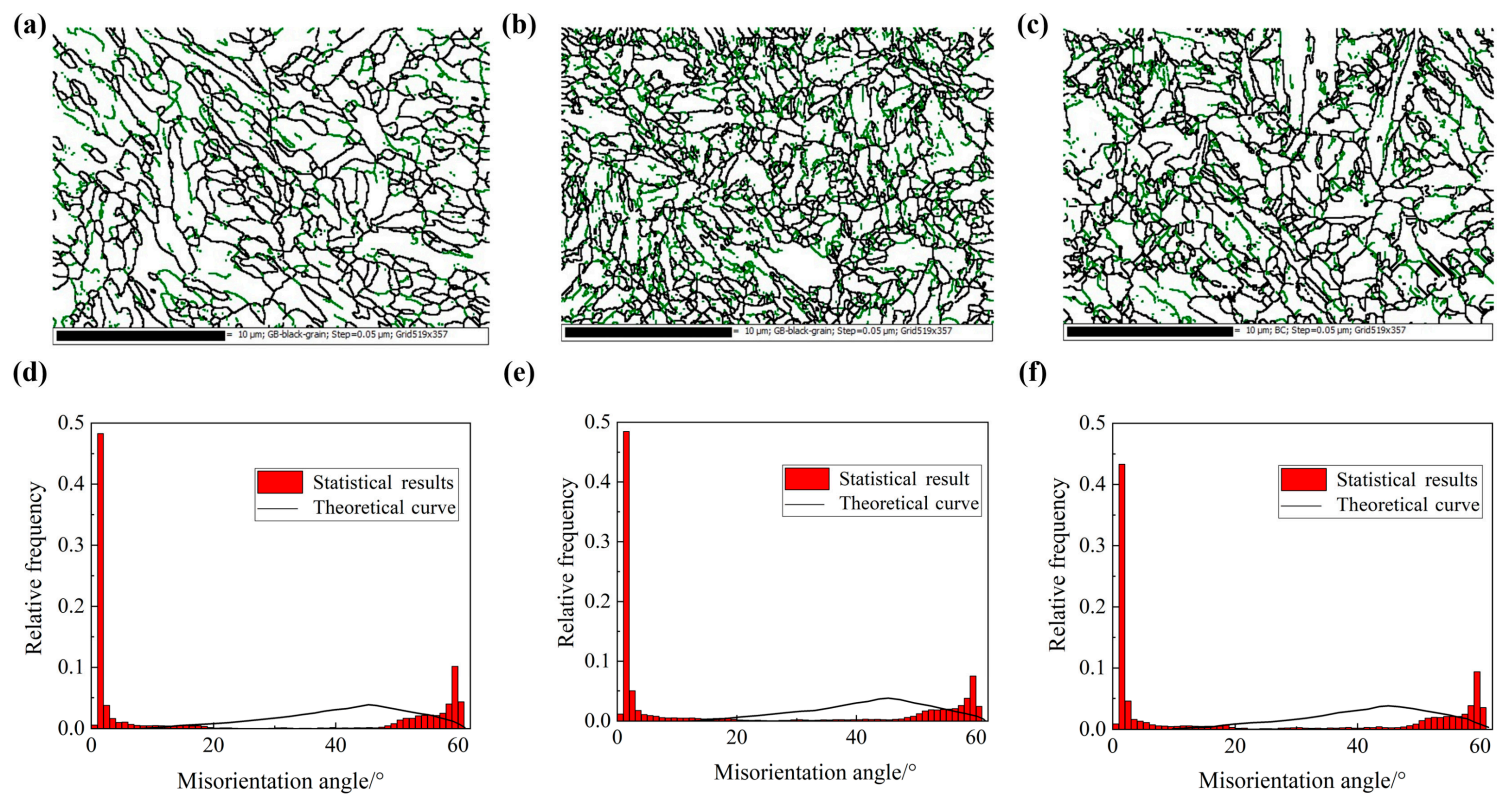
Grain boundary maps and misorientation statistical distribution of the test steel quenched at different heating temperatures: (**a**,**d**) 890 °C; (**b**,**e**) 910 °C; (**c**,**f**) 930 °C.

Figure 6 shows the grain boundary misorientation distributions of the test quenched steel at different austenitizing temperatures, where LAGBs and HAGBs are represented by the green and black lines, respectively. With increasing austenitizing temperature, the proportion of LAGBs and subgrains exhibits a rising trend, measuring 12.4% at 890 °C, 13.9% at 910 °C, and 14.0% at 930 °C. It is well established that LAGBs possess lower grain boundary energy and less curvature, and stronger cohesion with the matrix, rendering them less susceptible to crack initiation [31]. HAGBs tend to generate localized stress concentrations, which increase the material’s susceptibility to cracking and fracture, ultimately compromising its overall mechanical reliability and damage tolerance [32]. In hot-formed steels, an appropriate fraction of LAGBs is beneficial for accommodating dislocation accumulation, promoting strain homogenization, and impeding crack propagation to a certain extent, thereby contributing to enhanced ductility and toughness [33].

**Figure 7:**
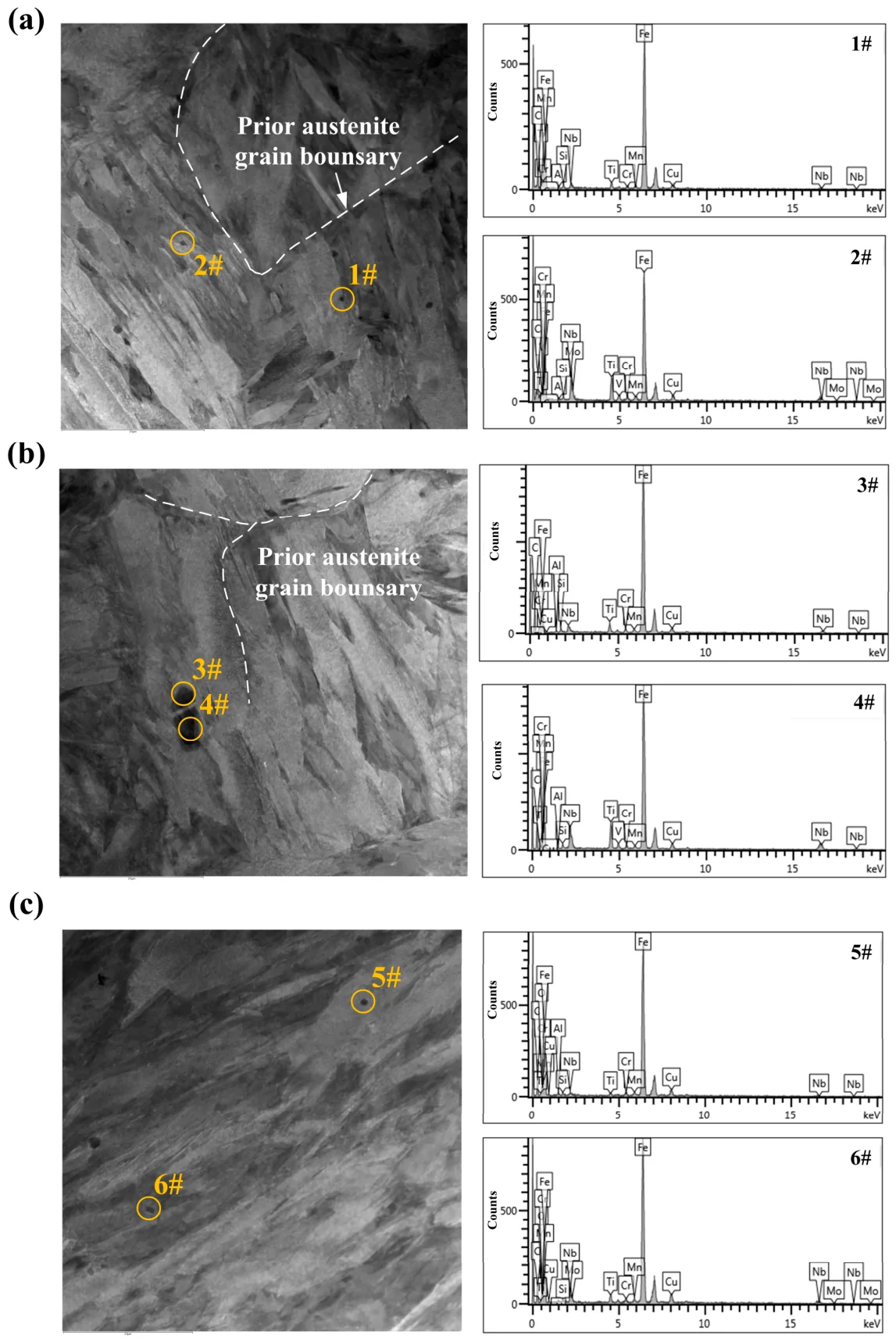
TEM maps and EDX spectroscopy of the test steel quenched at different heating temperatures: (**a**) 890 °C, (**b**) 910 °C, (**c**) 930 °C.

TEM is utilized to further investigate the microstructural characteristics of the quenched test steel. As shown in Figure 7, all samples exhibit a martensitic matrix with a substructure characterized by high-density dislocations, along with uniformly dispersed (Nb, Ti) C and (Nb, Ti, V) C complex precipitates ranging from 75 nm to 400 nm. As the austenite temperature increases, the average width of the martensite lath tends to decrease gradually. When the temperature is 890 °C, the average width of the martensite lath is 0.57 μm. With increasing austenitizing temperature, the average martensite lath width exhibits a decreasing trend, from 0.57 μm at 890 °C to 0.486 μm at 930 °C. Given that Nb, V, and Ti are transition metals with analogous physicochemical properties, their carbonitrides share similar crystal structures, which facilitates the formation of multi-component complex precipitates with enhanced thermal stability, rather than simple VC or NbC encapsulating TiN. Compared with single carbon-nitride phases, these multi-component solid solutions further reduce the system’s free energy due to increased mixing entropy. Consequently, these composite precipitates not only exert stronger grain-refinement and precipitation-strengthening effects but also possess appreciable hydrogen trapping capacity, which collectively contribute to improving the strength and toughness of hot-formed steel while mitigating the risk of hydrogen embrittlement.

**Figure 8:**
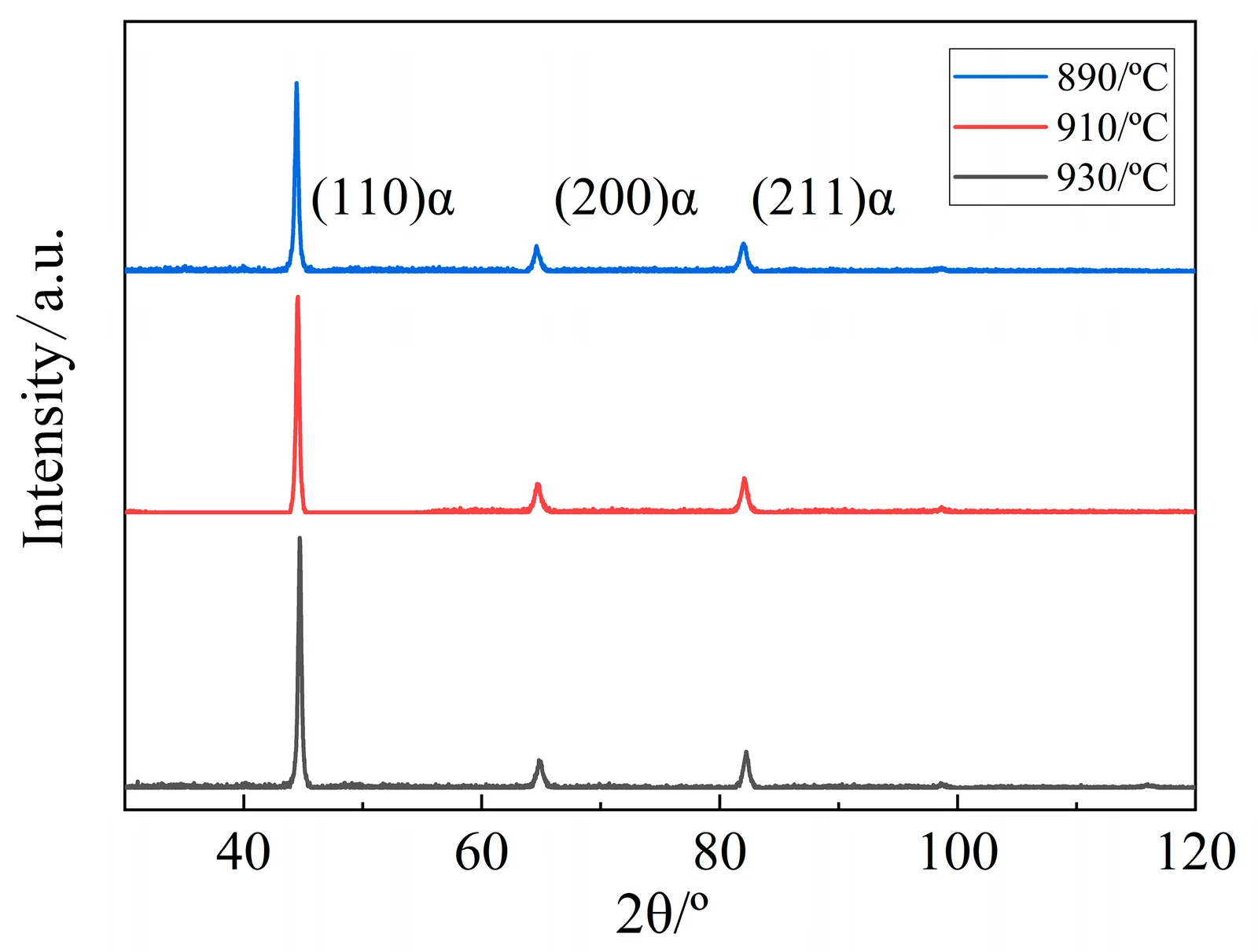
XRD patterns of the test steel quenched at different heating temperatures.

Figure 8 shows the XRD patterns of the test quenched steel at different austenitizing temperatures. For all austenitizing temperatures, the XRD patterns show that the microstructures are fully martensite, which show three α-phase diffraction peaks. Based on the Williamson–Hall model [34], the dislocation densities of the test quenched steel at different austenitizing temperatures of 890 °C, 910 °C and 930 °C are 0.54 × 10^16^ m^−2^, 0.76 × 10^16^ m^−2^, and 0.34 × 10^16^ m^−2^, respectively.

## 4. Numerical Simulation

**Figure 9:**
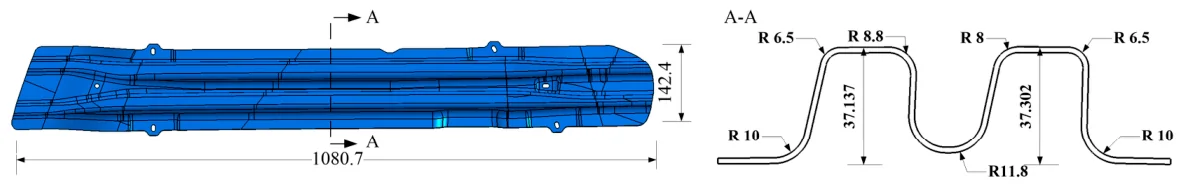
Geometry model of the anti-collision beam part (units: mm).

The anti-collision beam part model is shown in Figure 9, with overall dimensions of 1080.7 mm × 142.4 mm × 1.8 mm. This component features a “W-shaped” cross-section with a variable cross-sectional profile. Specifically, the section depth gradually decreases from the central A-A section toward both ends, exhibiting a smooth transition from deep to shallow.

**Figure 10:**
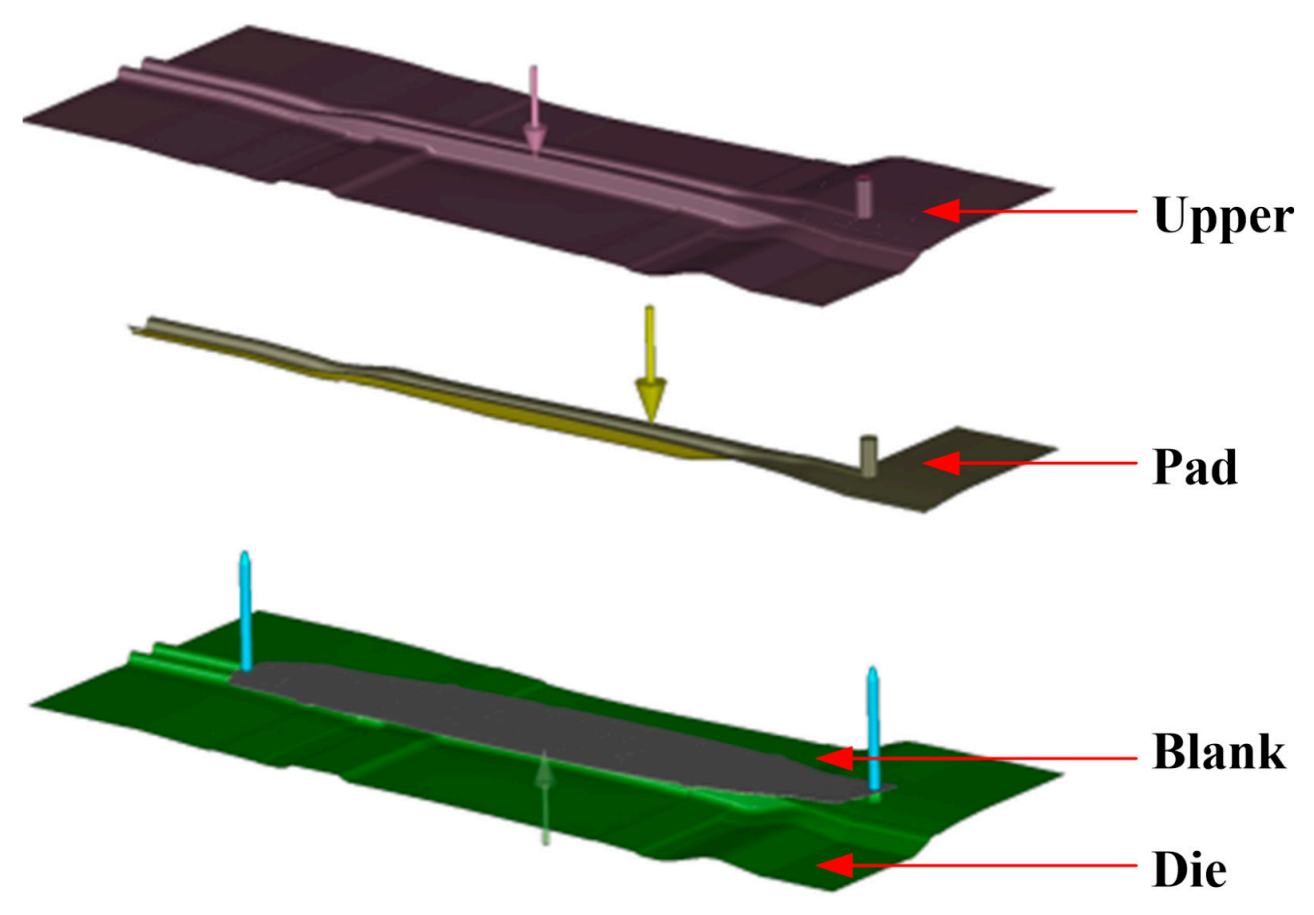
Finite element model in hot stamping numerical simulation.

Hot stamping simulation is conducted using Autoform R11 software, and the finite element model is shown in Figure 10. To balance geometric fidelity, computational accuracy, and efficiency, the maximum tool mesh size is set to 10 mm with a chord height of 0.05 mm. The maximum blank mesh size is 10 mm, the corner penetration tolerance is 0.22 mm, the maximum element normal angle is 22.5°, and the adaptive mesh refinement level is 6. The universal Coulomb friction model is employed in the numerical simulation, with the friction coefficient set to 0.45. During the blank transform from the furnace to the dies, heat loss from the blank occurs primarily via convection. The default heat transfer coefficient between the blank and air is applied: 0.02 mW· (mm^2^·k)^−1^ at 20 °C and 0.075 mW·(mm^2^·k)^−1^ at 950 °C. Once the blank makes contact with the dies, heat transfer is dominated by conduction, where the interfacial heat transfer coefficient (IHTC) depends on contact pressure and contact gap. The relationship between the contact gap and the IHTC factor follows the software’s default parameters, with an IHTC influence factor *f* = 0.15 at a contact gap if d_50%_ = 0.1.

## 5. Experiment

**Figure 11:**
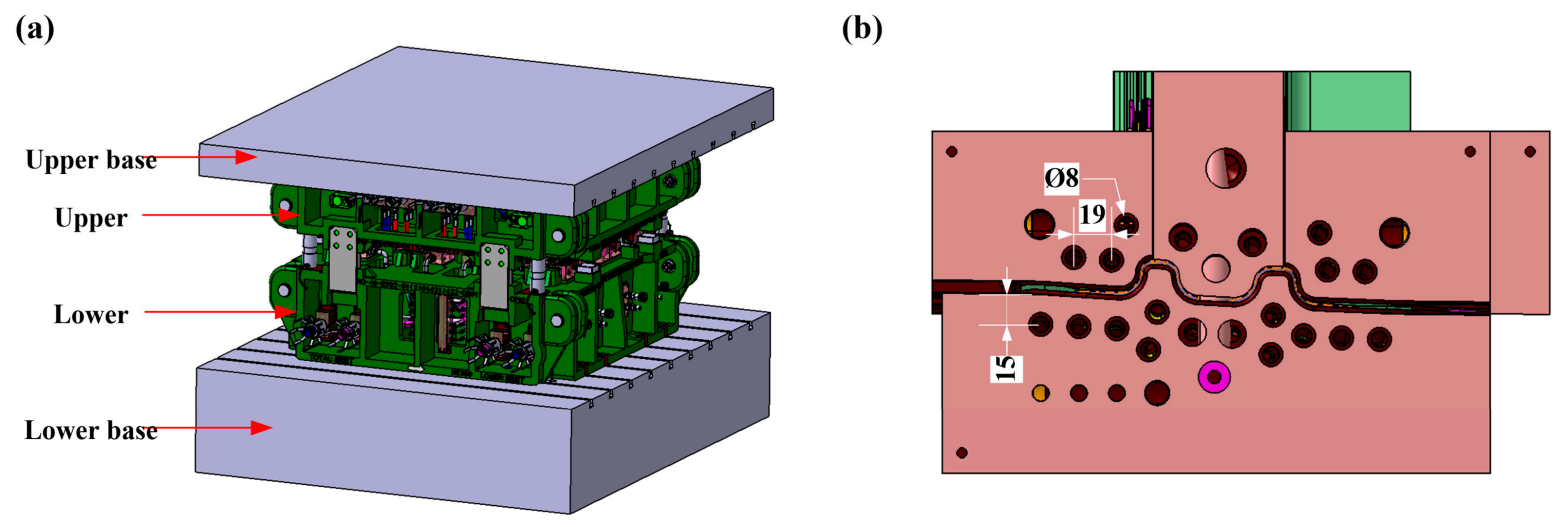
Die design: (**a**) assembly drawing, (**b**) schematic diagram of the cooling water channels.

The hot stamping die assembly illustrated in Figure 11 mainly comprises upper and lower die bases, upper and lower dies, a cooling system, a guiding and positioning system, and a blank-holding system. Each of the upper and lower dies is segmented into five inserts, with their parting lines deliberately offset to ensure effective cooling in the joint regions while preventing surface indentations on the formed blank. Conformal cooling channels with a diameter of 8 mm are machined within the inserts, following the contour of the part surface. These channels are arranged with a center-to-center spacing of 19 mm, and each channel center is positioned at a distance of 15 mm from the insert surface.

**Figure 12:**
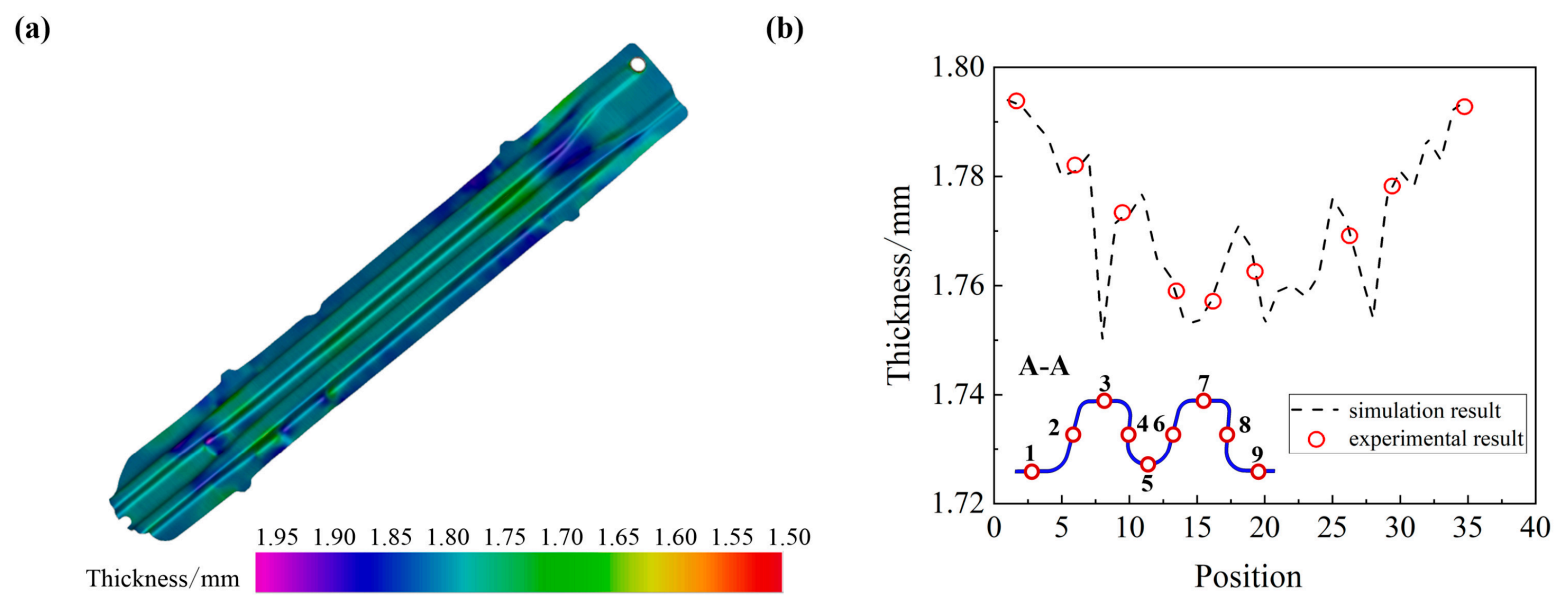
Thickness results of the door anti-collision beam part: (**a**) simulation results, (**b**) comparison between numerical simulation and measurement results.

Based on the hot stamping tests and numerical simulations, the blank is austenitized at 910 °C for 5 min and then transferred to the dies within 3 s~5 s. The forming speed of the hydraulic press is set to 70 mm/s, followed by quenching for 12 s under a clamping force of 1800 kN. Figure 12a presents the simulated thickness distribution of the anti-collision beam part. The minimum thickness is observed at the side wall of the reinforcing rib, indicating that this region undergoes substantial thermoplastic deformation during hot stamping. A comparison of the simulation and measurement thickness profiles along the A-A section reveals good agreement, thereby validating the reliability of the numerical simulation established in this study (Figure 12b).

**Figure 13:**
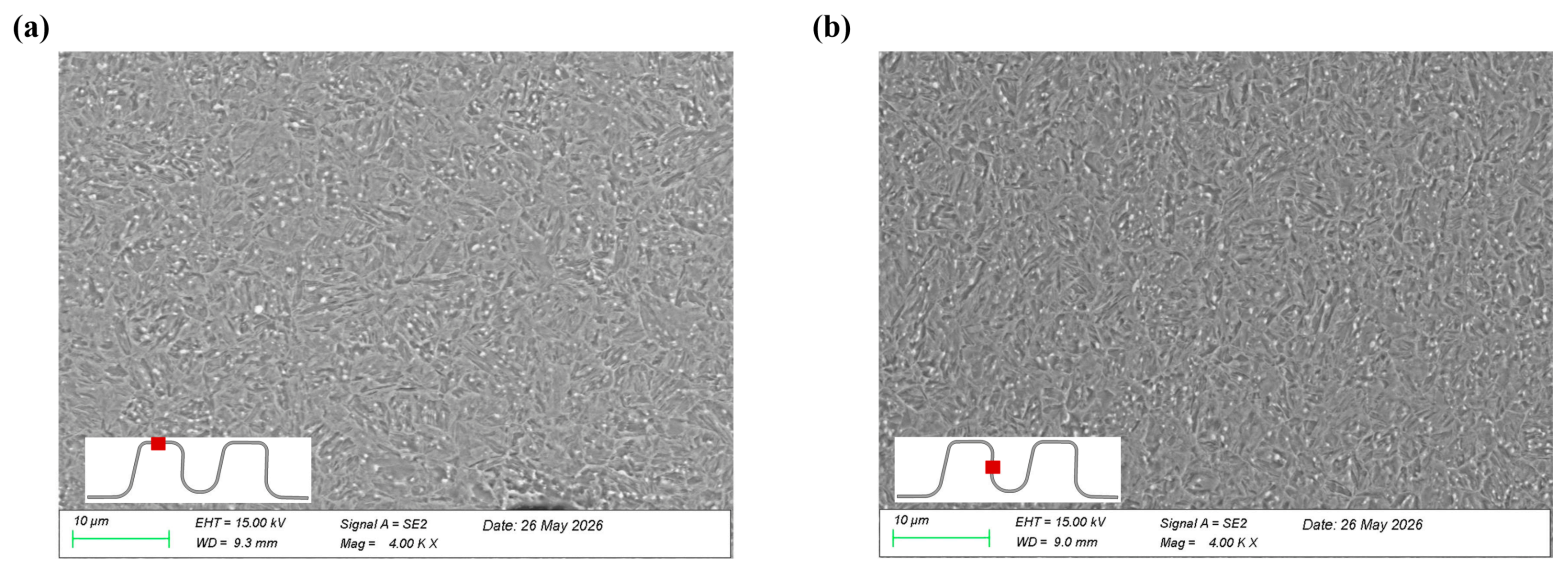
SEM images of the door anti-collision beam part: (**a**) top flat region, (**b**) side-wall region.

**Figure 14:**
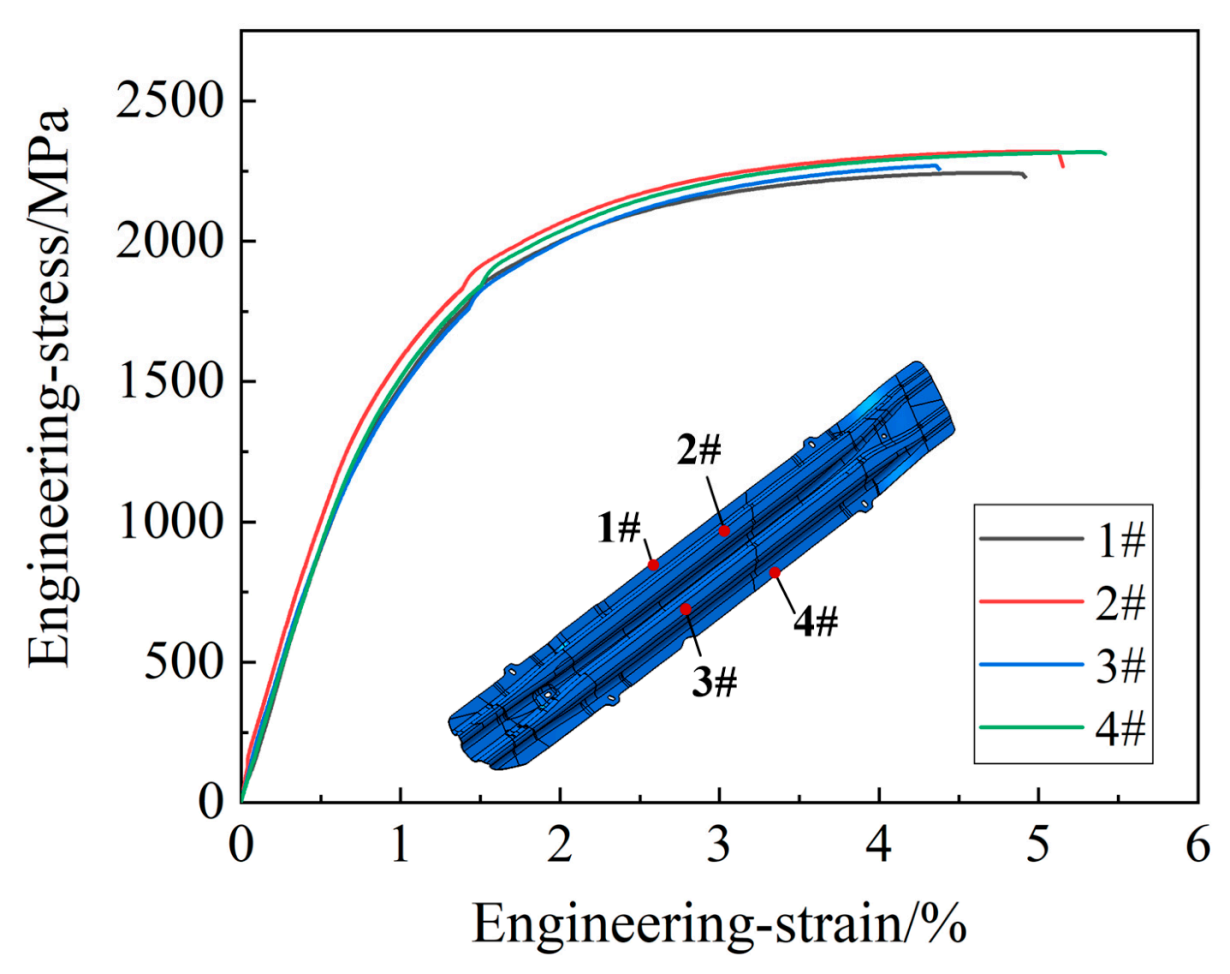
Engineering stress–strain curves of the door anti-collision beam part.

Figure 13 shows the SEM images of the reinforcing rib top flat region and the side-wall region of the anti-collision beam part. Both regions exhibit a fully martensitic microstructure, with fine Cr-based complex carbides uniformly dispersed. This confirms that a complete martensitic transformation is achievable within the present processing window. As a strong carbide-forming element, chromium readily combines with carbon to precipitate secondary-phase particles, thereby contributing to precipitation strengthening [35]. To further evaluate the mechanical properties of the part, tensile samples are extracted from both flanges and the reinforcing rib top flat region for testing. The engineering stress–strain curve results are shown in Figure 14. The tensile properties obtained at different locations are relatively comparable, indicating that the parts are uniformly quenching. The average *R_p_*_0.2_, *R_m_*_,_ and *A_t_* is 1480 MPa, 2287.9 MPa, and 4.96%, respectively.

## 6. Conclusions

(1) The CCT curve of the test steel is determined using the thermal dilatometry method, providing a theoretical foundation for establishing the hot stamping process. The *A*_c1_, *A*_c3_, *M*_s_, and *M*_f_ is 765.2 °C, 812.2 °C, 314 °C, and 190 °C, respectively, and the martensitic critical cooling rate is approximately 1 °C/s.
(2) The optimal heat treatment parameter identified in this study is austenitization at 910 °C for 5 min. Under this condition, the test steel achieves a tensile strength of 2201 MPa, a total elongation of 6%, with a refined austenite grain size of 7.47 μm, and the bending angle is about 42.7°.
(3) The addition of Nb, V, and Ti synergistically refines the prior austenite grains and promotes precipitation hardening. TEM analysis of the quenched samples reveals a martensitic matrix with a high-density dislocation structure, with uniformly distributed (Nb, Ti) C and (Nb, Ti, V) C complex precipitates ranging from 75 nm to 400 nm. These uniformly dispersed particles interact strongly with dislocations, effectively impeding their motion and thereby contributing substantially to the strengthening of the martensitic matrix.
(4) After hot stamping, the door anti-collision beam part exhibited a fully martensitic microstructure. The average mechanical properties of *R*_p0_._2_, *R*_m_, and *A*_t_ are 1480 MPa, 2287.9 MPa, and 4.96%, respectively. These results collectively validate the rationality of the cooling channel design and the effectiveness of the optimized process parameters employed in the hot stamping operation.

## Figures and Tables

**Table 1:** The chemical composition of the test steel (wt.%).

	C	Mn	Si	Cr	B	Mo	Nb	V	Ti	Fe
40MnB NbVTiMo	0.40	1.50	0.50	0.50	0.003	0.12	0.049	0.10	0.01	Bal.
	~	~	~	~	~	~	~	~	~	
	0.42	1.75	0.95	0.90	0.0035	0.20	0.052	0.12	0.05	

**Table 2:** Mechanical properties of the test steel after baking.

Temperature/°C	*R_p_*_0.2_/MPa	*R_m_*/MPa	*A_t_*/%	Bending Angle/°
890	1617 ± 35	2150 ± 28	5.3 ± 0.4	37.9 ± 1.5
910	1692 ± 30	2201 ± 25	6.0 ± 0.3	42.7 ± 1.2
930	1665 ± 32	2232 ± 22	5.5 ± 0.3	41.7 ± 1.3

## Data Availability

The original contributions presented in this study are included in the article. Further inquiries can be directed to the corresponding author.
